# Bioactive Flavonoid Glycosides and HPLC and UPLC Quantification of Commercial Astragali Complanati Semen

**DOI:** 10.3390/molecules25204762

**Published:** 2020-10-16

**Authors:** Jenny Chun-Ling Kuo, Li-Jie Zhang, Hung-Tse Huang, Chia-Ching Liaw, Zhi-Hu Lin, Min Liu, Yao-Haur Kuo

**Affiliations:** 1 Division of Chinese Internal Medicine, Center for Traditional Chinese Medicine, Chang Gung Memorial Hospital, Tao-Yuan 333, Taiwan; Jennykuo1115@gmail.com; 2 Division of Chinese Materia Medica Development, National Research Institute of Chinese Medicine, Ministry of Health and Welfare, Taipei 112, Taiwan; lijiezhang@hotmail.com (L.-J.Z.); kk49310953@nricm.edu.tw (H.-T.H.); liawcc@nricm.edu.tw (C.-C.L.); tiger77749@gmail.com (Z.-H.L.); 3 Department of Biochemical Science and Technology, National Taiwan University, Taipei 106, Taiwan; 4 Department of Biochemical Science and Technology, National Chiayi University, Chiayi 600, Taiwan; 5 Department of Life Science, Chinese Culture University, Taipei 113, Taiwan; lm@faculty.pccu.edu.tw; 6 Graduate Institute of Integrated Medicine, China Medical University, Taichung 404, Taiwan

**Keywords:** Astragali Complanati Semen, flavanol glycosides, UHPLC fingerprint, complanatuside, anti-oxidation

## Abstract

Eleven compounds, including nine known flavonoid glycosides (**1**–**4**, **6**–**8**, and **10**–**11**), one isoflavone glycoside (**5**), and a glansreginic acid (**9**), were isolated from the 80% ethanol extract of commercial Astragali Complanati Semen (ACS). All chemical structures were determined by spectroscopic analyses, including 1D and 2D NMR. Compounds **2**, **4**, **5**, **6**, **9**, and **10** were isolated and identified from the title plant for the first time. Biological evaluation revealed that all the isolates showed promising anti-NO production, and **1**, **2**, **3**, and **8** were more potent in antioxidant activity than vitamin E. The major peaks in the UPLC and HPLC profiles identified their chemical structures by comparing their retention time and UV spectra with those of the reference substances. Furthermore, nine of the eleven samples collected from North, Middle, and South regions of Taiwan possessed similar HPLC fingerprints and were identified as Astragali Complanati Semen, whereas the other two samples from southern Taiwan would be the adulterants due to the different fingerprinting patterns. In addition, an HPLC-UV method was employed to determine the content of target compound complanatuside (**11**) with good linear regression (R^2^ = 0.9998) for ACS in the Taiwanese market. Of the isolates, flavonol glycosides **1** and **3** were the major peaks in HPLC/UPLC, and showed more potent antioxidant and anti-NO production activities than that of **11**, revealing that these compounds can be the available agents for the quality control of ACS.

## 1. Introduction

Astragali Complanati Semen (Sha Yuan Ji Zi/Flastem milkvetech seed; ACS) is a traditional Chinese medicine, made from the ripe seeds of the perennial herbaceous plant *Astragalus complanatus* in the Leguminosae (Fabaceae). ACS has been used traditionally to tonify yang, strengthen the kidney, and consolidate kidney qi (the essential elements and vital energy for the body in Traditional Chinese Medicine theory), in result of stabilizing the essence such as seminal emission, spermatorrhea, enuresis, frequent urination, leukorrhagia and lumbar pain [1,2,3]. In the clinical setting, ACS serves as the main herb of decoction “Jin Suo Gu Jing Wan (Metal Lock Pill to Stabilize the Essence)” [4] or as an add-on therapy along with other Chinese medicine decoction for treating sexual dysfunction such as spermatorrhea, also known as premature ejaculation. Current therapeutic medications include selective serotonin reuptake inhibitor (SSRIs), serotonin and norepinephrine reuptake inhibitors (SNRIs), and phosphodiesterasetype 5 inhibitor (PDE5 inhibitor) [5]. However, when patients are treated with SSRIs, reduced libido and erectile dysfunction are common adverse side-effects. Interestingly, when treating erectile dysfunction with Chinese medicine, ACS is sometimes added into the formula for strengthening the yang, and patients experience less side effects, compared to that of SSRIs. Moreover, paroxetine, a kind of SSRI drug for treating premature ejaculation, showed a reducing NO production effect [6,7].

The main chemical constituents of Astragali Complanati Semen (ACS) are flavonoid glycosides (flavonol glycosides, isoflavone glycosides, and acylated flavonol glycosides) [8,9,10,11], fatty acids [12], and triterpene saponins [13], etc. Pharmacological investigations of the flavonoids yielded from ACS have demonstrated anti-liver fibrosis [14], antihypertensive effect [15], hypocholesterolemic effect [12], induction of apoptosis in human hepatocarcinoma SMMC-7721 cells [16], and protective effects on lung against paraquat injury [17], and on radiation-induced damages in mice [18]. In addition, the antioxidant activity has been shown to be highly correlated with content of phenolic fraction in the ACS [19]. These findings prompted us to investigate the potential secondary metabolites from ACS by employing antioxidant and anti-NO production assays.

Our subsequent phytochemical research on the 80% EtOH extract, which showed antioxidant activity by DPPH, has led to the isolation of nine known flavonol glycosides, myricetin 3-*O*-β-D-xylopyranosyl(1→2)-β-D-glucopyranoside (**1**) [20], myricetin 3-*O*-rutinoside (**2**) [21], myricetin 3-*O*-β-D-glucopyranoside (**3**) [22], quercetin 3-*O*-β-D-xylopyranosyl(1→2)-β-D-glucopyranoside (**4**) [22], laricitin 3-*O*-β-D-glucopyranoside (**6**) [23], kaempferol 3-*O*-β-D-xylopyranosyl(1→2)-β-D-glucopyranoside (**7**) [24], myricetin 3′-*O*-β-D-glucopyranoside (**8**) [11], kaempferol 3-*O*-β-D-glucopyranoside (**10**) [25], and complanatuside (**11**) [26], one isoflavone glycoside 3′-hydroxy-4′-methoxyisoflavone-7-*O*-β-D-glucopyranoside (**5**) [27], and a decadien derivative, (2*E*,4*E*)-8-hydroxy-2,7-dimethyl-decadien-(2,4)-disaeure-(1,10)-dioic acid (**9**) [28]. The structures of all isolated compounds were identified by extensive spectroscopic methods including MS and 2D NMR and comparison with the reported data. Compounds **2**, **4**, **5**, **6**, **8**, and **9** were isolated from the title plant for the first time. The HPLC and UPLC profiles were established, and characteristic peaks for the 11 major compounds were unambiguously confirmed. All the isolated flavonoid glycosides were further evaluated for antioxidant and anti-inflammatory activities herein.

It is reported that the seeds of *Astragalus sinicus* (Zi Yun Ying) and *Crotalaria pallida* (Zhu Shi Dou) were adulterating ACS in Taiwan and China. The quantity of flavonoids could be used as one of the useful indicators to comprehensively recognize and evaluate the efficacy of ACS. In fact, one flavonoid glycoside, complanatuside, has been used as the quality control component for ACS in China pharmacopoeia. Although ACS is a common traditional Chinese medicine in the market of Taiwan, the official remedy lacks a complanatuside assay item in the Taiwan pharmacopoeia.

In the present investigation, 11 batches of ACS samples collected from the North, Middle, and South regions of Taiwan were analyzed by HPLC and UPLC fingerprints. The isolated flavonoid compounds (**1**–**11**) from ACS were evaluated for antioxidant activity by DPPH and preliminary anti-inflammatory effects by the inhibition of LPS-induced NO production. Our findings should be useful in providing the available evidences for the quality control of ACS.

## 2. Results and Discussion

**Figure 1:**
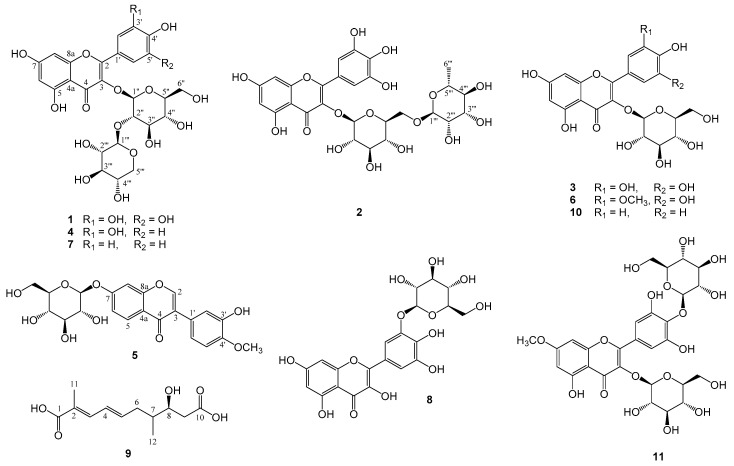
The chemical structures of compounds **1**–**11** isolated from Astragali Complanati Semen (ACS).

The 80% EtOH extract of ACS was suspended in H_2_O and further partitioned with *n*-hexane. The H_2_O layer was then loaded on Diaion HP-20, eluting with H_2_O, 25%, 50%, 75%, 95% ethanol, and EtOAc, successively. The fractions of 25% and 50% ethanol eluted exhibited potent antioxidant activity by the DPPH radical scavenging test (ED_50_ = 37.39 and 50.89 μg/mL, respectively). The HPLC profile indicated that all compounds in 25% ethanol fraction were also found in 50% ethanol fraction. The 50% ethanol fraction was subjected to column chromatography on Sephadex LH-20 and HPLC, and yielded thirteen compounds. The structures of isolated compounds (Figure 1) were identified by detailed spectroscopic analyses, using MS and 2D NMR, by comparing those of authentic samples and references. Eleven peaks in HPLC and UPLC fingerprints were further identified for their chemical structures. Compounds **2**, **4**, **5**, **6**, **8**, and **9** were isolated from ACS for the first time and their structural identification are as follows.

### 2.1. Structural Elucidation of the Isolated Compounds

**Figure 2:**
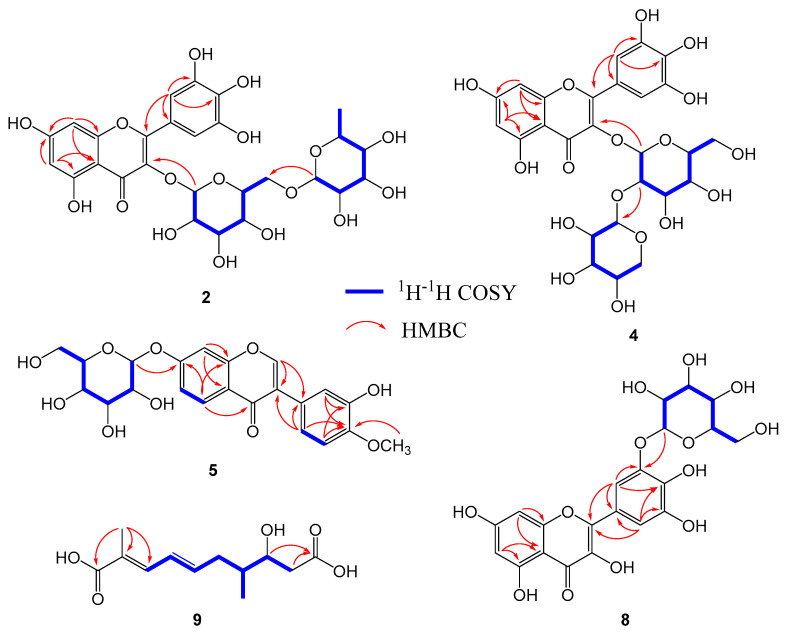
The key HMBC and ^1^H-^1^H COSY correlations of compounds **2**, **4**, **5**, **8**, and **9**.

Compound **2** was obtained as a yellowish powder with molecular formula C_27_H_30_O_17_, as established by positive ESIMS, in combination with NMR spectroscopic data. In the ^1^H NMR spectrum, four aromatic proton signals at δ_H_ 6.20 (1H, d, *J* = 2.0 Hz, H-6), 6.38 (1H, d, *J* = 2.0 Hz, H-8), and 7.28 (2H, s, H-2′, 6′), and two anomeric proton resonances for sugar moieties at 5.07 (1H, d, *J* = 7.2 Hz, H-1″) and 4.51 (1H, brs, H-1‴), were observed. The ^13^C NMR spectra (Table 1) showed resonance for 21 carbons differentiated by DEPT experiments, including one carbonyl (δ_C_ 179.4, C-4), four aromatic methines (δ_C_ 100.0 C-6; 94.9 C-8; 110.3 C-2′, 6′), ten quaternary SP2 signals (δ_C_ 159.4 C-2; 135.8 C-3; 105.6 C-4a; 163.0 C-5; 166.4 C-7; 158.5 C-8a; 122.3 C-1′; 146.4 C-3′, 5′; 138.0 C-4′), ten oxygenated methines (δ_C_ 102.4, 76.7, 78.2, 71.4, 77.2, 104.8, 72.1, 74.0, and 69.7), one oxygenated methylene (δ_C_ 68.6), and one methyl (δ_C_ 18.3). Those data indicated that compound **2** possessed a flavonol skeleton with a glucose and a rhamnose moieties [23]. The main planar structure of **2** was determined as myricetin, based on the detailed analysis of HMBC correlations (Figure 2), indicating that the A-ring was 5,7-dihydroxy substitution, and the B-ring was 3′,4′,5′-trihydroxy substitution located at C-2 of the C-ring. The long-range correlations of H-6″/C-1‴, H-1‴/C-6‴, and H-1″/C-3 further decided the linked positions of rhamnose to be C-6″ and the glucose to be C-3, respectively. The β- and α-anomeric configuration for glucose and rhamnose respectively, was judged by the coupling constants. Based on the above spectroscopic evidence, compound **2** was identified as myricetin 3-*O*-rutinoside [27].

Compound **4** presented as a yellowish powder, with a molecular formula of C_26_H_28_O_16_ determined from the ESIMS spectrum (*m*/*z* 619.1 [M + Na]^+^). The ^1^H and ^13^C NMR spectra of **4** indicated a flavone moiety with two sugars units. The ^1^H NMR spectrum showed resonances at δ_H_ 6.37 (1H, d, *J* = 2.0 Hz, H-8) and 6.18 (1H, d, *J* = 2.0 Hz, H-6), corresponding to two meta-coupling aromatic protons, and δH 7.63 (1H, brs, H-2′), 7.62 (1H, dd, *J* = 2.0, 8.4 Hz, H-6′), and 6.86 (1H, d, *J* = 8.4 Hz, H-5′), corresponding to a ABX system of aromatic protons, suggesting a quceretin skeleton for **4**. The ^13^C NMR spectrum (Table 2), together with two anomeric protons of β-glucose and xylose moieties in the ^1^H NMR spectrum, confirmed that **4** contained a quceretin with a xylopyranose and a glucopyranose moieties. As shown in the HMBC spectrum (Figure 2), a glucose and a xylose moieties were linked at C-3 and C-2″, respectively. With the above evidence, **4** was identified as quercetin 3-*O*-β-D-xylopyranosyl(1→2)-β-D-glucopyranoside [22].

Compound **5**, obtained as a yellowish powder, was assigned with the molecular formula C_22_H_22_O_10_, determined from ESIMS, ^13^C NMR, and DEPT spectroscopic data. The ^1^H and ^13^C NMR spectra (Table 1) showed resonance characteristics of isoflavone skeleton with a sugar moiety. The ^1^H NMR spectrum showed the characteristic signals, δ_H_ 8.33 (1H, s), for H-2 of the isoflavone framework, and two typical ABX system aromatic protons system for A-ring and B-ring in **5**. In addition, the signals for one aromatic methoxy group at δ_H_ 3.77 (3H, s) and an anomeric proton (δ_H_ 5.07, 1H, d, *J* = 7.2 Hz) of a β-glucose were observed. The positions of methoxy and glucose groups were further determined at C-4′ of B-ring and at C-7 of A-ring respectively, based on the HMBC spectrum (Figure 2). Therefore, compound **5** was identified as 3′-hydroxy-4′-methoxyisoflavone-7-*O*-β-D-glucopyranoside [27].

Laricitin 3-*O*-β-D-glucopyranoside (**6**) was isolated as a yellowish powder, and its ESIMS gave a quasimolecular ion at *m*/*z* 517.1 [M + Na]^+^ (C_22_H_22_O_13_Na). The ^1^H and ^13^C NMR spectra of **6**, as well as the substituted pattern, were similar to those of **3** (Table 2), except for the presence of an aromatic methoxy signal and the absence of a rhamnose moiety in **6**. The methoxy group was located at C-3′ based on HMBC correlations of H-2′/C-3′ and OCH_3_/C-3′ (Figure 2). Thus, compound **6** was identified as laricitin 3-*O*-β-D-glucopyranoside [23].

Compound **8** was obtained as a white powder. Its ESIMS (positive) showed a quasi-molecular ion peak at *m*/*z* 503.1 [M + Na]^+^, corresponding to the formula C_21_H_20_O_13_. The NMR spectra of **8** were very similar to those of **3**. A detailed comparison of the NMR spectra of compounds **3** and **8** showed the obviously different resonances at C-2 (**3**: δ_C_ 156.5; **8**: δ_C_ 146.6), C-3 (**3**: δ_C_ 133.7; **8**: δ_C_ 136.3), and C-1′ (**3**: δ_C_ 101.0; **8**: δ_C_ 102.9) in the ^13^C NMR spectrum (Table 1) and H-1″ (**3**: δ_H_ 5.43; **8**: δ_H_ 4.71) in the ^1^H NMR spectrum. The glucopyranose was further linked at C-3′ deduced from the HMBC spectrum; therefore, compound **8** was determined as myricetin 3′-*O*-β-D-glucopyranoside [11].

Compound **9** was obtained as a white powder. It showed a quasi-molecular ion peak at *m*/*z* 265.1 [M + Na]^+^ in ESIMS, corresponding to the formula C_12_H_18_O_5_, consistent with its ^1^H and ^13^C NMR data. The ^1^H-^1^H COSY spectrum showed the correlations of H-3/H-4/H-5/H-6/H-7/H-8/H-9 and H-7/CH_3_-11, allowing the establishment of structural fragments C-3 to C-9 and CH_3_-7. Based on the HMBC spectrum exhibiting H-3 to C-1 and CH_3_-2, H-8 to C-10, the planar structure of **9** was determined (Figure 2). The *E*-forms of the double bond were deduced from their coupling constants. Accordingly, compound **9** was identified as a glansreginic acid, (2*E*,4*E*)-8-Hydroxy-2,7-dimethyl-decadien-(2,4)-disaeure-(1,10)-dioic acid [28].

The others, compounds **1**, **3**, **7**, **10**, and **11**, were also isolated and identified by comparing their physical and spectroscopic data with those of authentic samples and references.

### 2.2. HPLC and UPLC Analysis of Astragali Complanati Semen

#### 2.2.1. The Optimization of Fingerprint Chromatographic Conditions

**Figure 3:**
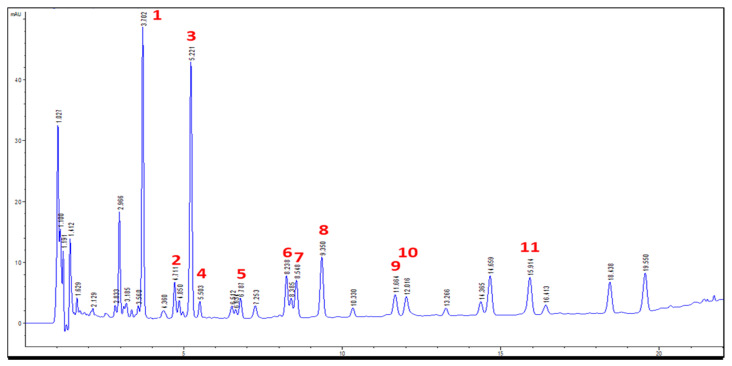
The UPLC profile of Astragali Complanati Semen (254 nm).

In order to obtain a good resolution within a short analysis time, the composition of mobile phase was optimized. Acidic mobile phase was used in order to suppress the ionization of phenolic hydroxyl groups of flavonoids. In this study, water and acetonitrile were chosen as the mobile phase for the low column pressure. We found that many interfering compounds were present in the plant material. Thus, the gradient elution program was carried out to separate these components in samples. Various acids, such as 0.1% ethylic acid, formic acid, and phosphoric acid in acetonitrile (B) and H_2_O (A) (*v*/*v*), were evaluated. Acetonitrile and water both containing 0.1% formic acid were chosen as the mobile phases because most components could be resolved under this condition. The elution program was as follows: 0–10 min, 15% B, 10–70 min, 15–28.8% B, 70–80 min, 28.8–38% B, 80–90 min, 38–100% B for HPLC, and 0–18 min, 15–21% B, 18–22 min, 21–32% B, 22–25 min, 32–100% for UPLC. Checking the UV spectra of the components recorded from 220 to 370 nm, 254 nm was finally selected for monitoring. As shown in Figure 3, 11 reference compounds in the real sample or standard mixture could be separated well within 20 min in UPLC, and good peak shapes were observed for all peaks.

**Figure 4:**
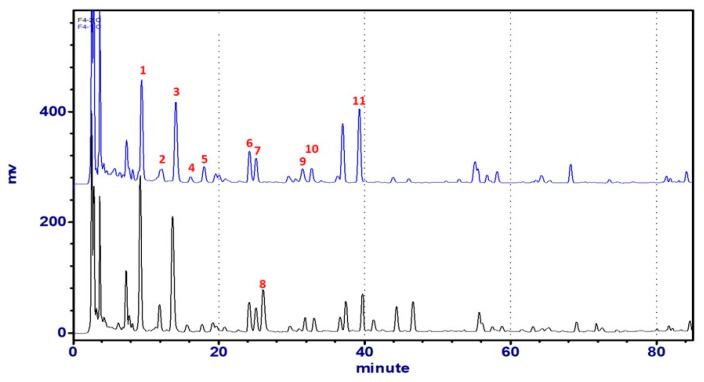
The HPLC-UV profile of Astragali Complanati Semen (254 nm). Blue line: The extract dissolved in MeOH was freshly prepared. Black line: The extract dissolved in MeOH was stored at room temperature for 3 weeks.

We studied the HPLC fingerprint profile (Figure 4) of the 80% EtOH crude extract of ACS by using the HPLC-UV method and identified 11 main peaks by comparing the retention times with the reference compounds isolated from the title plant. The mobile phase consisted of water (A) and acetonitrile (B) with 0.1% formic acid, using a gradient program of 15%–15%–28.8%–100% (B) in 0–10–70–75 min. In the fingerprint chromatograph, the retention times for compounds **1** to **11** were shown: **1**: 8.6, **2**: 11.0, **3**: 12.6, **4**: 13.1, **5**: 14.6, **6**: 22.6, **7**: 23.4, **8**: 24.4, **9**: 30.3, **10**: 31.0, and **11**: 37.9 min, respectively.

#### 2.2.2. The Optimization of Complanatuside Quantification Analysis Chromatographic Conditions

According to the China Pharmacopoeia, the chromatographic conditions of complanatuside quantification analysis were set as follows. The mobile phase system was 0.1% H_3_PO_4_ aq. (A) and acetonitrile (B), with the gradient program of 0–17 min, 21% B, 17–17.1 min, 21–100% B. The UV detection wavelength was 254 nm. The column temperature was kept at 25 °C. The flow rate was 1.0 mL/min and the injection volume was 20 μL. These conditions assured that complanatuside (Rt: 13.9 min) in the real sample was baseline separated from the neighbor peaks.

#### 2.2.3. Optimization of Extraction Conditions

**Figure 5:**
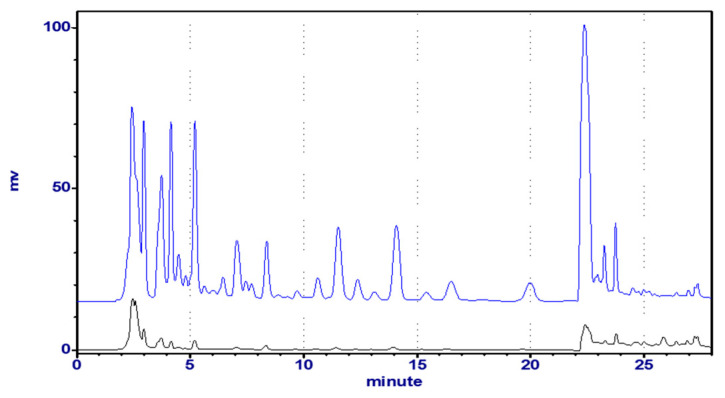
HPLC chromatograph comparison of extraction times (Blue: the first extract, black: the second extract).

The extraction solvent and extraction times were optimized in order to achieve satisfactory extraction efficiency. The extraction efficiency of 40%, 60%, 80%, 95% ethanol, 40%, 60%, 80% pure methanol, and H_2_O were evaluated. The 40% ethanol and 60% methanol extract had the same yield of complanatuside, but the sample dissolved in methanol seems unstable, especially the target compound complanatuside, which decreased after 3 weeks (Figure 4). Thus, the 40% ethanol was selected as the most suitable solvent for extraction. Extraction methods containing ultrasonic and reflux extraction were tested and compared. Although the extraction efficiency was approximated, the former was easier and simpler to perform than the latter. The extraction times were also compared, resulting in 97% and 3% yield of complanatuside for the first and second extract, respectively (Figure 5). Therefore, ultrasonication for 30 min in 40% ethanol extracted one time was chosen as the optimal extraction condition for the following experiments.

#### 2.2.4. Chromatographic Analyses of Astragali Complanati Semen

**Figure 6:**
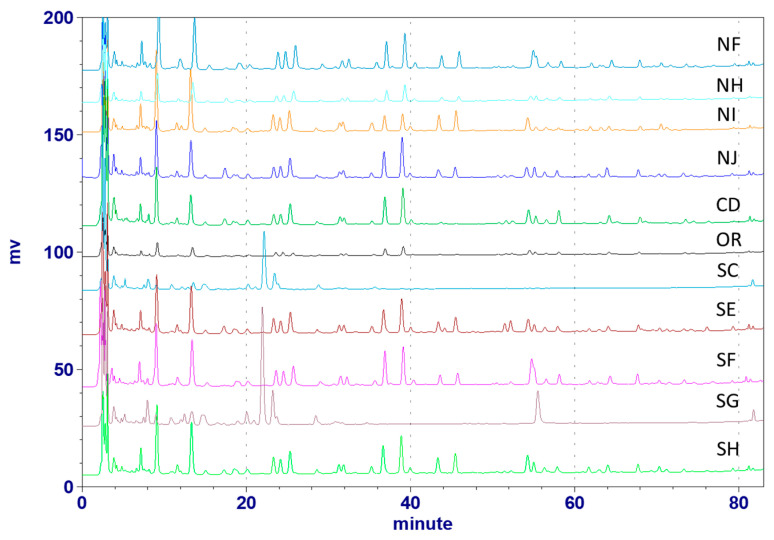
HPLC profiles of Astragali Complanati Semen samples collected from the North, Middle, and south of Taiwan (254 nm).

We studied the HPLC profile of the 40% ethanol extracts of 11 samples collected from the North, Middle, and South Chinese herbal medicine companies of Taiwan. As shown in Figure 6, the 6 samples from the Northern and the Middle of Taiwan had similar secondary metabolite patterns but the contents depended on the source. In all samples collected from the Southern Taiwan, 3 samples (SE, SF, SH) showed similar secondary metabolite patterns, but samples SC and SG were different in in their HPLC profiles. The results identified nine samples collected as ACS, whereas two other samples from the Southern area would be the adulterants due to the different HPLC patterns.

#### 2.2.5. Validation of Quantitative Analysis Method

The complanatuside calibration curve linear equation was y = 18,221.2x − 3915.1 and showed good linear regression (R_2_ = 0.9998) within test ranges (1.9 to 150 μg/mL). The LOD (S/N = 3), LOQ (S/N = 10), precision (inter-day and intraday, 60.0, 15.0, 1.9 μg/mL), injection precision (15 μg/mL), reproducibility (n = 5), and stability (n = 5) for complanatuside are summarized in Table 2. The RSD values of the injection, intra-day, inter-day, and stability variations were less than 2.99%, except that of reproducibility, which was 4.25%. The results of the recovery test are summarized in Table 3, and the RSD was 3.30%. Therefore, the HPLC-UV method was precise, accurate, and sensitive enough for quantitative analysis of complanatuside in ACS.

#### 2.2.6. Quantitative Determination of Astragali Complanati Semen

This HPLC assay method was subsequently applied to quantify complanatuside in ACS obtained from different Chinese herbal medicine stores distributed in the North, Middle, and South of Taiwan. Their contents are listed in Table 4. The contents varied from 0.062% to 0.134%, which were acceptable according to both of the 2010 China pharmacopoeia and Taiwan Herbal Pharmacopeia (0.06%), except the samples of SC and SG (Table 4).

In the present study, a simple, accurate, and efficient HPLC method was developed to evaluate the quality of commercial ACS by establishing the complanatuside quantification method. The results demonstrate that this method is accurate, reproducible, and could be readily employed as a suitable quality control method for ACS.

We established the HPLC fingerprint of ACS, and identified 11 compounds in the profile. Ten of eleven compounds were flavonoids, suggesting that flavonoids were the main components in the ACS. The collected 11 commercial ACS samples showed similar morphological features. The HPLC/UPLC methods as described above can distinguish true ACS from the false ones among 11 commercial samples collected from three different areas of Taiwan (North area: NF, NH, NI, NJ; Middle area: CD, CR; South area: SC, SE, SF, SG, SH). The fingerprint indicated that samples SC and SG were pseudo, and the other nine samples were genuine ACS. Samples SC and SG were further identified as the seeds of *A. adsurgens*, based on the referenced HPLC profile. Therefore, the developed HPLC-UV method can be readily used in quality assurance, as well as inspection of adulteration of ACS.

### 2.3. The Isolates and Fractions of the Antioxidation and Anti-NO Production Activities

**Figure 7:**
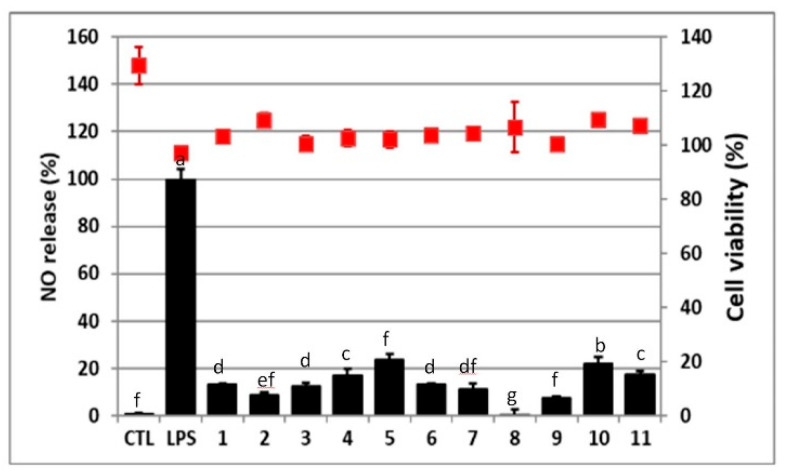
Anti-NO production activities (black column) and cell viability (red square) of the isolated compounds **1**–**11** (20 μg/mL). Cells were treated with LPS (1 μg/mL) or in combination with tested isolates (20 μg/mL) for 24 h. All values are presented as mean ± SD (n = 3). CTL: Control group. LPS: Treatment with LPS. The significant difference was analyzed by one-way ANOVA with Duncan’s test in SPSS. Each group used different letters (a–f) to show statistical significances (*p* < 0.05) in anti-NO activity.

All isolated flavonoid glycosides and compound **9** were evaluated for antioxidant and anti-inflammatory activities. In the antioxidant assay, compounds **1**, **2**, **3**, and **8** showed more potency than the positive control vitamins C and E (Table 5). Crude extract and partial purified fractions were evaluated for antioxidant activity, and 80ED25 fraction showed a scavenging DPPH radical effect (ED_50_ = 37.39 ± 0.33 μg/mL). In this study, all tested compounds showed the promising potency on anti-NO production, and compound **8** was the most inhibitory (Figure 7). Although the isolated compounds **1**, **2**, **3**, and **8** all exhibited obvious inhibition both on antioxidant and anti-NO production, **1** and **3** were selected as the potential target compounds based on their much bigger peaks than those of **2** and **8** in HPLC/UPLC profiles of ACS.

In previous reports, ACS extracts exhibited potent activities on DPPH, superoxide radical, hydroxyl radical, and Fe^2+^-induced lipid peroxidation [19,29]. In an in vivo study, ACS extracts were reported to improve ROS effect by increasing the blood’s important antioxidant enzymes, superoxide dismutase and glutathione peroxidase [30]. Another study revealed that the total flavonoids extracted from ACS had comprehensive effects on immune response, including anti-NO, anti-ROS, lymphocyte activation, and critical immune index, including thymus and spleen, in an aging mouse model [31]. Many flavonoids and their derivatives yielded from the other plants also possessed antioxidant and anti-NO production activities [32]. Our results suggested that the flavonoid glycosides of ACS played an important role in antioxidant and anti-NO production activities, especially compounds **1**, **2**, **3**, and **8**. In addition, herbal medicines used in treating premature ejaculation and sexual dysfunction activities have been shown to contain flavonoid derivatives as their major ingredients [33,34] which supports the therapeutic option for choosing ACS since ACS are also high in flavonoid content.

## 3. Experimental

### 3.1. General

ESIMS data were performed on mass spectrometer (Quattro Ultima, Waters Corp. Milford, CT, USA). 1D and 2D NMR spectra were performed on a Bruker NMR spectrometer (Unity Plus 400 MHz, Bruker Corp, Billerica, MA, USA) using MeOD-d_4_ and DMSO-d_6_ as solvents for measurement. Diaion HP20 (Mitsubishi Chemical, Tokyo, Japan) and Sephadex LH-20 (GE Healthcare, Chicago, IL, USA) were used for column chromatography. HPLC separations were performed on a Shimadzu LC-20A (Shimadzu, Kyoto, Japan) series apparatus (Pump: LC-20AT; UV detector: SPD-20A), equipped with a 250 × 20 mm preparative Cosmosil 5C_18_-ARII column. HPLC fingerprint plot was done on a Shimadzu 10A (Shimadzu, Japan) series system equipped with a LC-20AT pump, SPD-10Avp UV-Vis detector, 7715i manual injector with 20 μL sample loop, Biotech 2003 degasser (Biotech, model 2003, Onsala, Sweden), SCL 10Avp system controller, and SISC–LAB32 software (Scentific Information Service Corporation, Taipei, Taiwan), with a Cosmosil 5C18-ARII column (5 μm, 4.6 × 250 mm, Nacalai Tesque, INC., Tokyo, Japan).

### 3.2. Plant Material

The dried seed of Astragalus Complanatus (Astragali Complanati Semen; ACS) for the isolation was purchased from Tianshun Chinese herbal medicine pharmacy (Taipei, Taiwan) in March 2014. A voucher specimen (NRICM 2014034601) was identified by Dr. Yao-Haur Kuo and deposited in the National Research Institute of Chinese Medicine, Taipei, Taiwan. The eleven commercial samples were collected from the North (Taipei, Taiwan), Middle (Taichung, Taiwan), and South (Kaohsiung, Taiwan) of Taiwan in March 2014.

### 3.3. Extraction and Isolation

Astragali Complanati Semen (ACS, 6.0 kg) was extracted with 80% EtOH (40 L) three times at 50 ℃. The crude extracts were combined and concentrated under reduced pressure. The residue was suspended in H_2_O, and then partitioned with n-hexane, obtaining an n-hexane layer (80EH, 121.9 g). Then, the H_2_O layer was loaded on a Diaion HP-20 column, and successively eluted with 100% H_2_O (80ED0, 188.2 g), 25% aq. EtOH (80ED25, 80.2 g), 50% aq. EtOH (80ED50, 70.2 g), 75% aq. EtOH (80ED75, 17.8 g), 95% aq. EtOH (80ED95, 2.8 g), and 100% EtOAc (80EDEA, 1.7 g). The part III (50% aq. EtOH, 80ED50, 10 g) was separated by Sephadex LH-20 column eluting with 70% MeOH to yield compounds **3** (0.64 g), **8** (0.55 g), **11** (0.81 g), and sub-fraction 80ED50125 (3.2 g). The subfraction 80ED50125 (0.8 g) was further purified by reverse phase preparative HPLC (Cosmosil 5C_18_ AR-II column, 250 × 20 mm, 18% acetonitrile (ACN) in H_2_O, 10.0 mL/min), and the same column and flow rate were used in the next preparative HPLC experiments to afford compounds **1** (25.1 mg), **4** (24.4 mg), and **7** (64.4 mg). The 80ED50 fraction (10.2 g) was subjected to an ODS flash chromatography column, eluting with gradient mobile phase (10% to 50% MeOH in H_2_O, *v*/*v*), to give three fractions (80ED5001 ~ 80ED5003). Fraction 80ED5001 was subjected to Sephadex LH-20, eluting with 70% MeOH, to afford compound **5** (0.21 g) and subfractions 80ED50011 and 80ED50013. Subfraction 80ED50011 was purified by reverse phase preparative HPLC (20% ACN) to afford compound **9** (102.1 mg). Sub-fraction 80ED50013 was also purified by reverse phase preparative HPLC (18% ACN) to afford compounds **2** (44.1 mg), **6** (73.9 mg), and **10** (55.4 mg).

### 3.4. Spectroscopic Data

*Myricetin 3-O-β-D-xylopyranosyl(1→2)-β-D-glucopyranoside (1)*: Yellowish powder; ^1^H NMR (in MeOD-*d*_4_, 400 MHz) δ_H_: 7.26 (2H, s, H-2′,6′), 6.36 (1H, brs, H-8), 6.18 (1H, brs, H-6), 5.48 (1H, d, *J* = 7.2 Hz, Glc H-1), 4.77 (1H, d, *J* = 7.2 Hz, Xyl H-1); the ^13^C NMR data (in MeOD-*d*_4_, 100 MHz) are shown in Table 1; positive ESIMS *m*/*z* 635.1 [M + Na]^+^, C_26_H_28_O_17_.

*Myricetin 3-O-rutinoside (2)*: Yellowish powder; ^1^H NMR (in MeOD-*d*_4_, 400 MHz) δ_H_: 7.28 (2H, s, H-2′,6′), 6.38 (1H, d, *J* = 2.0 Hz, H-8), 6.20 (1H, d, *J* = 2.0 Hz, H-6), 5.07 (1H, d, *J* = 7.2 Hz, Glc H-1), 4.51 (1H, brs, Rha H-1); the ^13^C NMR data (in MeOD-*d*_4_, 100 MHz) are shown in Table 1; positive ESIMS *m*/*z* 649.1 [M + Na]^+^, C_27_H_30_O_17_.

*Myricetin 3-O-β-D-glucopyranoside (3)*: Yellowish powder; ^1^H NMR (in DMSO-*d*_6_, 400 MHz) δ_H_: 12.60 (1H, s, OH-5), 7.16 (2H, s, H-2′,6′), 6.37 (1H, d, *J* = 2.0 Hz, H-8), 6.18 (1H, d, *J* = 2.0 Hz, H-6), 5.43 (1H, d, *J* = 7.6 Hz, Glc H-1); the ^13^C NMR data (in DMSO-*d*_6_, 100 MHz) are shown in Table 1; positive ESIMS *m*/*z* 503.1 [M + Na]^+^, C_21_H_20_O_13_.

*Quercetin 3-O-β-D-xylopyranosyl(1→2)-β-D-glucopyranoside (4)*: Yellowish powder; ^1^H NMR (in MeOD-d4, 400 MHz) δ_H_: 7.63 (1H, brs, H-2′), 7.62 (1H, dd, *J* = 2.0, 8.4 Hz, H-6′), 6.86 (1H, d, *J* = 8.4 Hz, H-5′), 6.37 (1H, d, *J* = 2.0 Hz, H-8), 6.18 (1H, d, *J* = 2.0 Hz, H-6), 5.48 (1H, d, *J* = 7.6 Hz, Glc H-1), 4.74 (1H, d, *J* = 7.2 Hz, Xyl H-1); the ^13^C NMR data (in MeOD-*d*_4_, 100 MHz) are shown in Table 1; positive ESIMS *m*/*z* 619.1 [M + Na]^+^, C_26_H_28_O_16_.

*3*′*-Hydroxy-4*′*-methoxyisoflavone-7-O-β-D-glucopyranoside (5)*: Yellowish powder; ^1^H NMR (in DMSO-*d*_6_, 400 MHz) δ_H_: 9.12 (1H, s, OH-3′), 8.33 (1H, s, H-2), 8.03 (1H, d, *J* = 8.8 Hz, H-5), 7.20 (1H, d, *J* = 2.0 Hz, H-8), 7.14 (1H, dd, *J* = 8.8, 2.0 Hz, H-6),7.02 (1H, brs, H-2′), 6.94 (2H, s, H-5′, 6′), 5.07 (1H, d, *J* = 7.2 Hz, Glc H-1), 3.77 (3H, s, OCH3); the ^13^C NMR data (in DMSO-*d*_6_, 100 MHz) are shown in Table 1; positive ESIMS *m*/*z* 469.1 [M + Na]^+^, C_22_H_22_O_10_.

*Laricitin 3-O-β-D-glucopyranoside (6)*: Yellowish powder; ^1^H NMR (in MeOD-*d*_4_, 400 MHz) δ_H_: 7.49 (1H, d, *J* = 1.6 Hz, H-2′), 7.28 (1H, d, *J* = 1.6 Hz, H-6′), 6.34 (1H, brs, H-8), 6.16 (1H, brs, H-6), 5.36 (1H, d, *J* = 7.2 Hz, Glc H-1), 3.90 (3H, s, OCH_3_-3′); the ^13^C NMR data (in MeOD-*d*_4_, 100 MHz) are shown in Table 1; positive ESIMS *m*/*z* 517.1 [M + Na]^+^, C_22_H_22_O_13_.

*Kaempferol 3-O-β-D-xylopyranosyl(1→2)-β-D-glucopyranoside (7)*: Yellowish powder; ^1^H NMR (in MeOD-*d*_4_, 400 MHz) δ_H_: 8.06 (2H, d, *J* = 8.8 Hz, H-2′, 6′), 6.88 (2H, d, *J* = 8.8 Hz, H-3′, 5′), 6.38 (1H, d, *J* = 1.6 Hz, H-8), 6.18 (1H, d, *J* = 1.6 Hz, H-6), 5.46 (1H, d, *J* = 7.6 Hz, Glc H-1), 4.74 (1H, d, *J* = 6.8 Hz, Xyl H-1); the ^13^C NMR data (in MeOD-*d*_4_, 100 MHz) are shown in Table 1; positive ESIMS *m*/*z* 603.1 [M + Na]^+^, C_26_H_28_O_15_.

*Myricetin 3*′*-O-β-D-glucopyranoside (8)*: Yellowish powder; ^1^H NMR (in DMSO-*d*_6_, 400 MHz) δ_H_: 12.41 (1H, s, OH-5), 7.50 (1H, d, *J* = 2.0 Hz, H-6′), 7.48 (1H, d, *J* = 2.0 Hz, H-2′), 6.44 (1H, d, *J* = 2.0 Hz, H-8), 6.17 (1H, d, *J* = 2.0 Hz, H-6), 4.71 (1H, d, *J* = 7.2 Hz, Glc H-1); the ^13^C NMR data (in DMSO-*d*_6_, 100 MHz) are shown in Table 1; positive ESIMS *m*/*z* 503.1 [M + Na]^+^, C_21_H_20_O_13_.

*(2E,4E)-8-Hydroxy-2,7-dimethyl-decadien-(2,4)-disaeure-(1,10)-dioic acid (9)*: White powder; ^1^H NMR (in MeOD-*d*_4_, 400 MHz) δ_H_: 7.17 (1H, d, *J* = 11.6 Hz, H-3), 6.44 (1H, dd, *J* = 11.6, 14.8 Hz, H-4), 6.08 (1H, td, *J* = 7.2, 14.8 Hz, H-5), 2.45 (1H, m, H-6), 2.10 (1H, m, H-6), 3.85 (1H, m, H-7), 2.52 (1H, dd, *J* = 3.6, 14.8 Hz, H-9), 2.35 (1H, dd, *J* = 9.6, 14.8 Hz, H-9), 1.89 (3H, s, CH3-11), 0.90 (3H, s, CH_3_-12); the ^13^C NMR data (in MeOD-*d*_4_, 100 MHz) δ_C_: 172.1 (C-1), 126.3 (C-2), 140.1 (C-3), 128.8 (C-4), 142.6 (C-5), 37.0 (C-6), 40.3 (C-7), 72.9 (C-8), 40.1 (C-9), 176.0 (C-10), 12.6 (C-11), 15.8 (C-12); positive ESIMS *m*/*z* 265.1 [M + Na]^+^, C_12_H_18_O_5_.

*Kaempferol 3-O-β-D-glucopyranoside (10)*: Yellowish powder; ^1^H NMR (in DMSO-*d*_6_, 400 MHz) δ_H_: 12.56 (1H, brs, OH-5), 8.01 (2H, d, *J* = 8.4 Hz, H-2′, 6′), 6.87 (2H, d, *J* = 8.4 Hz, H-3′, 5′), 6.42 (1H, brs, H-8), 6.19 (1H, brs, H-6), 5.41 (1H, d, *J* = 7.2 Hz, Glc H-1); the ^13^C NMR data (in DMSO-*d*_6_, 100 MHz) are shown in Table 1; positive ESIMS *m*/*z* 471.1 [M + Na]^+^, C_21_H_20_O_11_.

*Complanatuside (11)*: Yellowish powder; ^1^H NMR (in DMSO-*d*_6_, 400 MHz) δ_H_: 12.52 (1H, s, OH-5), 8.13 (2H, d, *J* = 8.8 Hz, H-2′, 6′), 7.14 (2H, d, *J* = 8.8 Hz, H-3′, 5′), 6.74 (1H, d, *J* = 2.0 Hz, H-8), 6.37 (1H, d, *J* = 2.0 Hz, H-6), 5.46 (1H, d, *J* = 7.6 Hz, 3-Glc H-1), 5.00 (1H, d, *J* = 7.2 Hz, 4″-Glc H-1), 3.84 (3H, s, OCH3); the ^13^C NMR data (in DMSO-*d*_6_, 100 MHz) are shown in Table 1; positive ESIMS *m*/*z* 647.1 [M + Na]^+^, C_28_H_32_O_16_.

### 3.5. Sample Preparation for HPLC and UPLC Assays

All raw samples of ACS were ground into powder and filtered through a 65-mesh sieve (0.25 mm). An accurately weighed 0.5 g powder sample was placed into a 50 mL Centrifuge tube with stopper, and 25 mL 40% ethanol was added exactly to the centrifuge tube, and then ultrasonic extracted for 30 min (500 W, 40 kHz). The resultant mixture was adjusted to the original weight with extraction solvent, and the supernatant was removed through a 0.45 or a 0.22 μm membrane filter before HPLC and/or UPLC injection, respectively.

### 3.6. HPLC Fingerprint Assay

The reference compounds **1**–**11** were prepared and purified as mentioned above. Formic acid (98–100%) was purchased from Merck (Darmstadt, Germany) and ethanol (95%) from Echo Chemical Co. Ltd. (Toufen, Taiwan). Acetonitrile (LC grade) was purchased from Macron fine chemicals (Avantor performance material, Gliwice, Poland). Milli-Q ultra-pure water (Millipore, Q-gard 1/Quantum EX, Burlington, MA, USA) was used throughout the study. Apparatus and conditions: the HPLC profile was performed on a Shimadzu 10A series system equipped with a LC-20AT pump, SPD-10Avp UV-Vis detector, 7715i manual injector with 20 μL sample loop, Biotech 2003 degasser (Biotech, model 2003, Sweden), SCL 10Avp system controller, and SISC software (Scientific Information Service Corporation, Taipei, Taiwan). The chromatographic separation was carried out on a Cosmosil 5C_18_-AR-II (5 μm, 250 × 4.6 mm i.d., Japan). The binary gradient elution system consisted of 0.1% formic acid aq. (A) and 0.1% formic acid acetonitrile (B), and the HPLC profile separation was achieved using the following gradient: 0–10 min, 15% B; 10–70 min, 15–28.8% B; 70–80 min, 28.8–38% B; 80–90 min, 38–100% B. Preparation of sample solution: the 80% EtOH extract was dried under vacuum, then accurately weighed to about 200 mg and dissolved in 80% EtOH, in a 10 mL volumetric flask. The sample solution was filtered through a 0.45 μm filter (Millipore) before use, and the injective volume was 20 μL. The UV detection wavelength was 254 nm. The column temperature was kept at room temperature and the flow rate was 1.0 mL/min.

### 3.7. Complanatuside Quantification Analysis

Standard stock solution of complanatuside (**11**) was prepared with 40% ethanol. From the stock solution, a series of working standard solutions were prepared by dilution with 40% ethanol to the proper concentrations and stored at 4 °C. The mobile phase system for **11** quantification analysis was used as 0.1% H_3_PO_4_ aq. (A) and acetonitrile (B), with the gradient program of 0–17 min, 21% B; 17–17.1 min, 21–100% B. The UV detection wavelength was 254 nm. The column temperature was kept at 25 °C. The flow rate was 1.0 mL/min and the injection volume was 20 μL. The UPLC fingerprint was performed on an Agilent 1200 Infinity Series liquid chromatography system (Santa Clara, CA, USA) equipped with a binary solvent delivery system (1290 Bin Pump), a diode array detector (1290 DAD), a column temperature controller (1290 TCC), and an autosampler (1290 Sampler). Chromatographic data were recorded and processed using Agilent chromatographic work station software. The UPLC experiment was carried out on an Agilent Poroshell 120, EC-C_18_ Column (150 × 3.0 mm, 2.7 μm), which was protected by an EC-C_18_ guard column (5.0 × 3.0 mm, 2.7 μm). The mobile phase condition was similar to that of HPLC, the binary gradient elution system consisted of 0.1% formic acid aq. (A) and 0.1% formic acid acetonitrile (B), and the UPLC fingerprint separation was achieved using the following gradient: 0-18 min, 15–21% B; 18–22 min, 21–32% B; 22–25 min, 32–100% B. The UV detection wavelength was 254 nm, the injection volume was 2 μL, and the flow rate was 0.6 mL/min.

### 3.8. Validation Procedure

The calibration curve was established by plotting the peak area (y) versus the concentration (x) of each analysis, using working standard solutions of complanatuside, and contained five different concentrations performed in triplicate. The stock solution was further diluted to a series of concentrations with 40% EtOH to determine the limit of detection (LOD) and limit of quantitation (LOQ) on the basis of the signal-to-noise ratio (S/N) of 3 and 10, respectively. The injection precision was determined consequently by replicate injection of the same standard solution (15.0 μg/mL), five times. The intra-day precision was determined by analyzing three replicates of each standard solution (60.0, 15.0, and 1.9 μg/mL) within one day, while inter-day variation was determined on three consecutive days. In order to evaluate the repeatability of the developed method, sample solutions prepared from five parallel samples (0.5 g, SF sample) were determined within one day. To choose one from the sample solutions, the complanatuside concentrations were analyzed at 0, 3, 6, 12, and 24 h to evaluate the stability of the solution at 25 °C. The recovery test was carried out by the standard addition method. The standard stock solution (150 μg/mL, 2.5 mL, accurately) was added into the sample (0.5 g, SF sample), and then extracted, processed, and quantified, and five parallel samples were prepared. The average recovery was calculated by the following equation: recovery (%) = (amount found − amount contained)/amount added × 100%, and RSD (%) = (SD/mean) × 100%. Samples from different regions of Taiwan were prepared as described above. An aliquot (20 μL) of the filtrate was directly subjected to HPLC analysis. Each sample was determined in triplicate. Chromatographic peaks of the samples were quantified by the external standard method.

### 3.9. Scavenging Activity of DPPH Radical Assay

The antioxidative activity of the isolates on DPPH (1,1-diphenyl-2-picrylhydrazyl)-free radical was measured using the method of Rangkadilok et al., with minor modifications [35]. An aliquot of each sample (120 μL, 200–5 μg/mL) or vitamin E (40–5 μg/mL) was mixed with 30 μL of 0.75 mM of DPPH methanol solution in a 96-well microplate. The mixture was shaken vigorously with an orbital shaker in the dark at room temperature for 30 min and the absorbance was measured at 517 nm with an ELISA reader. Methanol was used as the negative control by replacing the sample in the reaction solution. The DPPH radical scavenging activities of the test samples were compared to the negative control and vitamins C and E as the positive controls. The final results were performed as the concentrations of ED_50_, which is the concentration of each sample required to give 50% of the absorbance shown by the negative control.

### 3.10. LPS-Induced NO Production of Anti-Inflammatory Assays

The macrophage cell line RAW 264.7 was obtained from ATCC (Rockville, MD, USA) and cultured in DMEM containing 10% heat-inactivated fetal calf serum, 100 U/mL penicillin, and 100 μg/mL streptomycin, and grown at 37 ℃ with 5% CO_2_ in fully humidified air. Cells were plated at a density of 2 × 10^5^ cells/well in a 96-well culture plate and stimulated with lipopolysaccharide (LPS, 1 μg/mL) in the presence or absence of different concentrations of tested compounds (20 μg/mL) for 18 h, simultaneously. All compounds were dissolved in DMSO and further diluted with sterile PBS and sterilized via a 0.2 μm filter. Nitrite (NO_2_^−^) accumulation in the medium was used as an indicator of NO production which was measured by adding Griess reagent (1% sulfanilamide and 0.1% naphthylenediamine in 5% phosphoric acid). NaNO_2_ was used to generate a standard curve, and nitrite production was determined by measuring optical density at 550 nm [36]. All experiments were performed in triplicate. NO production by LPS stimulation was designated as 100% for each experiment. A well-known *i*NOS inhibitor, quercetin, was employed as a positive control (IC_50_ = 7.87 ± 0.14 μM).

## 4. Conclusions

In our study, flavonoid glycosides were the main components of Astragali Complanati Semen (ACS) and proved to have potent antioxidation and anti-NO effects. Our established HPLC-UV method can be used to determine the content of flavonol glycoside complanatuside (**11**) with good linear regression (R^2^ = 0.9998) for commercial ACS in the Taiwanese market. Using our established HPLC/UPLC methods, nine out of eleven commercial samples collected from three areas of Taiwan were reliably identified to be genuine ACS.

Biological evaluation revealed that flavonol glycosides **1**, **2**, **3**, and **8** isolated from ACS had more potent antioxidant activity (by DPPH) than the positive control vitamins C and E, and all the isolated flavonol derivatives showed promising effects on anti-NO production. By comparing all the isolates, both myricetin 3-*O*-β-D-xylopyranosyl(1→2)-β-D-glucopyranoside (**1**) and myricetin 3-*O*-β-D-glucopyranoside (**3**) showed the most obvious peaks in HPLC/UPLC. The results, together with **1** and **3** exhibiting more potent antioxidant and anti-NO production activities than that of **11**, the quality control component for ACS in China and Taiwan pharmacopoeia, suggested that flavonol glycosides **1** and **3** can be the available compounds for the quality control of ACS.

## Figures and Tables

**Table 1:** ^13^C NMR spectroscope data of compounds **1**–**8** and **10**–**11**.

No.	1 ^a^	2 ^a^	3 ^b^	4 ^a^	5 ^b^	6 ^a^	7 ^a^	8 ^b^	10 ^b^	11 ^b^
2	158.4	159.4	156.5	158.4	153.8	158.3	158.3	146.6	156.7	156.6
3	135.3	135.8	133.7	135.1	124.6	135.6	135.0	136.3	133.5	134.2
4	179.5	179.4	177.6	179.6	175.1	179.3	179.6	176.1	177.8	177.9
4a	105.8	105.6	104.1	105.8	118.7	105.7	105.7	103.3	104.3	105.3
5	163.1	163.0	161.4	163.1	127.3	162.9	163.1	164.2	161.5	161.1
6	99.7	100.0	98.8	99.8	115.9	99.8	99.9	98.5	99.1	98.2
7	165.8	166.4	164.3	165.9	161.7	165.9	166.1	164.2	164.4	165.5
8	94.5	94.9	93.6	94.6	103.7	94.7	94.7	93.90	94.0	92.6
8a	158.4	158.5	156.5	158.3	157.3	158.6	158.5	156.4	156.7	156.2
1′	122.1	122.3	120.2	123.2	123.9	121.9	122.8	121.3	121.2	123.8
2′	109.9	110.3	108.7	116.1	116.6	106.7	135.0	107.7	115.5	116.0
3′	146.5	146.4	145.6	146.1	146.2	148.9	116.2	146.6	131.3	130.9
4′	137.9	138.0	136.8	149.7	147.9	138.7	161.5	137.5	160.2	159.5
5′	146.5	146.4	145.6	117.3	112.2	146.1	116.2	146.6	131.3	130.9
6′	109.9	110.3	108.7	123.4	120.1	111.1	135.0	110.9	115.5	116.0
OCH_3_					55.9	57.0				56.4
1″	101.0	102.4	101.0	100.9	100.2	103.8	100.8	102.9	101.2	101.0
2″	82.2	75.7	74.1	82.3	73.3	75.9	82.4	73.5	74.5	74.4
3″	76.9	78.2	76.7	77.0	76.6	78.1	77.1	76.1	767	76.7
4″	70.9	71.4	70.0	71.0	69.9	71.4	71.1	69.9	70.2	70.1
5″	78.2	77.2	77.7	78.2	77.4	78.4	78.2	77.4	77.7	77.7
6″	62.3	68.6	61.2	62.4	60.9	62.5	62.4	60.9	61.1	61.0
1‴	105.2	104.8		105.4			105.5			100.1
2‴	74.8	72.1		74.9			75.0			73.4
3‴	78.3	72.1		78.4			78.4			76.6
4‴	70.5	74.0		71.0			71.0			69.8
5‴	66.5	69.7		66.6			66.7			77.2
6‴		17.9								60.8

^a^ Data were measured at 100 MHz in MeOD-*d*_4_; ^b^ Data were measured at 100 MHz in DMSO-*d*_6_.

**Table 2:** Method validation of complanatuside quantification by HPLC.

Method Validation Item	Complanatuside (11)	
	Concentration (μg/mL)	RSD %
Injection precision (n = 5)	15.0	1.35
Intraday (n = 3)	60.0	0.80
	15.0	1.35
	1.9	1.00
Inter-day (n = 9)	60.0	1.18
	15.0	2.01
	1.9	2.99
Reproducibility (n = 5)	SF (0.5 g)	4.25
Stability (n = 5)	SF (0.5 g)	1.04
LOD (S/N = 3 μg/mL, n = 1)	0.019	-
LOQ (S/N = 10 μg/mL, n = 1)	0.038	-

**Table 3:** Analysis of the recovery of complanatuside (n = 5).

No.	Original (mg)	Addition (mg)	Detection (mg)	Recovery (%)
1	0.6001	0.389	1.0051	104.12
2	0.6006	0.389	0.9870	99.34
3	0.6013	0.389	0.9983	102.07
4	0.6002	0.389	0.9930	100.99
5	0.6001	0.389	1.0208	108.14
Average	0.6005	0.389	1.0009	102.93
RSD (%)	0.085	-	1.30	3.30

**Table 4:** The complanatuside content of 11 batches of commercial Astragali Complanatus Semen.

Samples	Complanatuside (%)	Samples	Complanatuside (%)
NF	0.106 ± 0.004 *	SC	0 ^#^
NH	0.098 ± 0.001 *	SE	0.115 ± 0.004 **
NI	0.095 ± 0.002 *	SF	0.120 ± 0.002 **
NJ	0.134 ± 0.002 **	SG	0^#^
CD	0.116 ± 0.006 *	SH	0.109 ± 0.003 **
OR	0.062 ± 0.002	THP/CHP	0.06

THP: Taiwan Herbal Pharmacopeia; CHP: Pharmacopoeia of the People’s Republic of China. One-way analysis of variance (ANOVA) followed by Dunnett’s test were used to analyze the data. The statistical analysis was performed with SPSS (* *p* < 0.01, ** *p* < 0.001). ^#^ not detected.

**Table 5:** Antioxidant activity of DPPH scavenging ^a*^ for the extract, fractions, and the isolates **1**–**11**.

The Extract	Removal Effect (200 μg/mL, %)	ED_50_ (μg/mL)	Compound	Removal Effect (100 μg/mL, %)	ED_50_ (μM)
80E	53.34 ± 1.26	180.42 ± 5.22	**1**	98.93 ± 0.01 ^a^	14.34 ± 0.59 ^ab^
80EH	10.67 ± 1.17	(-) ^b*^	**2**	98.39 ± 0.00 ^a^	16.59 ± 0.85 ^b^
80ED0	13.12 ± 0.72	(-) ^b*^	**3**	99.73 ± 0.13 ^a^	19.27 ± 0.46 ^b^
80ED25	99.23 ± 0.33	37.39 ± 0.33	**4**	94.54 ± 0.13 ^b^	39.36 ± 1.26 ^d^
80ED50	96.20 ± 3.00	50.89 ± 0.13	**5**	58.77 ± 0.13 ^c^	200.06 ± 0.63 ^g^
80ED75	57.31 ± 1.18	164.29 ± 3.58	**6**	97.23 ± 0.13 ^ab^	33.18 ± 1.64 ^c^
80ED95	64.61 ± 1.64	139.74 ± 3.39	**7**	1.97 ± 1.01 ^f^	(-) ^c*^
80EDEA	34.29 ± 0.62	(-) ^b*^	**8**	99.55 ± 0.13 ^a^	14.77 ± 0.44 ^a^
			**9**	5.37 ± 3.79 ^e^	(-) ^c*^
			**10**	31.57 ± 1.39 ^d^	(-) ^c*^
			**11**	0.15 ± 0.28 ^g^	(-) ^c*^
Vitamin C	- ^d*^	4.34 ± 0.28	Vitamin C	-	24.64 ± 1.58 ^d^
Vitamin E	-	12.67 ± 0.09	Vitamin E	-	29.42 ± 0.21 ^f^

^a*^ All values are presented as mean ± SD (n = 3). ^b*^ ED_50_ value > 200 μg/mL. ^c*^ ED_50_ value > 200 μM. ^d*^ not tested. The significant difference was analyzed by one-way ANOVA with Duncan’s test in SPSS. Each group used different letters (a–g) to show statistical significance at *p* < 0.05.

## Abbreviations

SMMC-7721	human hepatocarcinoma
1D/2D NMR	1Dimensional/2Dimensional Nuclear Magnetic Resonance
EtOH	Ethanol
DPPH	2,2-Diphenyl-1-picrylhydrazyl
CDCl_3_	Chloroform
MS	Mass Spectrometry
ESIMS	Electrospray Ionization Mass Spectrometry
NMR	Nuclear Magnetic Resonance
LPS	Lipopolysaccharides
UPLC	Ultra Performance Liquid Chromatography
HPLC	High Performance Liquid Chromatography
ED_50_	50% Effective Dose
IC_50_	50% Inhibitory Concentration
DEPT	Distortionless Enhancement by Polarization Transfer
HMBC	Heteronuclear Multiple Bonds Correlation
COSY	Correlation Spectroscopy
Rt	Retention time
UV	Ultraviolet spectroscopy
IR	Infrared spectroscopy
HPLC-UV	High Performance Liquid Chromatography with Ultraviolet Detector
MeOH	Methanol
LOD	Level of Development
LOQ	Limit Of Quantitation
RSD	Relative Standard Deviation
SD	Standard Deviation
ANOVA	Analysis Of Variance
ELISA	Enzyme-Linked Immunosorbent Assay

## References

[1] Committee on Chinese Medicine and Pharmacy (2019). Taiwan Herbal Pharmacopoeia.

[2] Pharmacopoeia Commission of People’s Republic of China (2010). Pharmacopoeia of the People’s Republic of China.

[3] Ng Y.-F., Tang P.C.-T., Sham T.-T., Lam W.-S., Mok D.K., Chan S.-W. (2014). Semen Astragali Complanati: An ethnopharmacological, phytochemical and pharmacological review. J. Ethnopharmacol..

[4] Yu D., Yang J., Analyses N. (1999). Analytical Chemistry Manual VII Fascicle.

[5] Martin C., Nolen H., Podolnick J., Wang R. (2017). Current and emerging therapies in premature ejaculation: Where we are coming from, where we are going. Int. J. Urol..

[6] Zhang D., Cheng Y., Wu K., Ma Q., Jiang J., Yan Z. (2019). Paroxetine in the treatment of premature ejaculation: A systematic review and meta-analysis. BMC Urol..

[7] Angulo J., Peiró C., Sanchez-Ferrer C.F., Gabancho S., Cuevas P., Gupta S., Sáenz de Tejada I. (2001). Differential effects of serotonin reuptake inhibitors on erectile responses, NO-production, and neuronal NO synthase expression in rat corpus cavernosum tissue. Br. J. Pharm..

[8] Cui B., Kinjo J., Nakamura M., Nohara T. (1991). A novel acylated flavonoid glycoside from *Astragalus complanatus*. Tetrahedron Lett..

[9] Cui B.L., Lu Y.R., Wei L.X. (1989). Studies on chemical constituents of *Astragalus complanatus* R. BR. Yao Xue Xue Bao.

[10] Cui B., Nakamura M., Kinjo J., Nohara T. (1992). Structures of three new acylated flavonol glycosides from *Astragalus complanatus* R. BR. Chem. Pharm. Bull..

[11] Cui B., Nakamura M., Kinjo J., Nohara T. (1993). Chemical constituents of Atragali semen. Chem. Pharm. Bull..

[12] Sham T.T., Zhang H., Mok D.K.W., Chan S.W., Wu J.H., Tang S.Y., Chan C.O. (2017). Chemical anaylsis of Astragali Complanati Semen and Its Hypocholesterolemic effect using serum metabolomics based on gas chromatography-mass spectrometry. Antioxidants.

[13] Cui B., Sakai Y., Takeshita T., Kinjo J., Nohara T. (1992). Four new oleanene derivatives from the seeds of *Astragalus complanatus*. Chem. Pharm. Bull..

[14] Liu C.Y., Gu Z.L., Zhou W.X., Guo C.Y. (2005). Effect of *Astragalus complanatus* flavonoid on anti-liver fibrosis in rats. World J. Gastroenterol..

[15] Xue B., Li J., Chai Q., Liu Z., Chen L. (2008). Effect of total flavonoid fraction of *Astragalus complanatus* R. Brown on angiotensin II-induced portal-vein contraction in hypertensive rats. Phytomedicine.

[16] Zhang Z., Dong Y., Li X., Peng L. (2014). Total flavonoids from *Astragalus complanatus* attenuates lung injury following paraquat poisoning in rats through inhibiting excessive endoplasmic reticulum stress and c-Jun *N*-terminal kinase pathway. Chin. Crit. Care Med..

[17] Hu Y.W., Liu C.Y., Du C.M., Zhang J., Wu W.Q., Gu Z.L. (2009). Induction of apoptosis in human hepatocarcinoma SMMC-7721 cells in vitro by flavonoids from *Astragalus complanatus*. J. Ethnopharmacol..

[18] Qi L., Liu C.Y., Wu W.Q., Gu Z.L., Guo C.Y. (2011). Protective effect of flavonoids from *Astragalus complanatus* on radiation induced damages in mice. Fitoterapia.

[19] Zhang Q.A., Fan X.H., Zhang Z.Q., Li T., Zhu C.P., Zhang X.R., Song W. (2013). Extraction, antioxidant capacity and identification of Semen Astragali Complanati (Astragalus complanatus R. Br.) phenolics. Food Chem..

[20] Zhang Y., He W., Li C., Chen Q., Han L., Liu E., Wang T. (2013). Antioxidative flavonol glycosides from the flowers of *Abelmouschus manihot*. J. Nat. Med..

[21] Lu Y., Sun Y., Foo L.Y., McNabb W.C., Molan A.L. (2000). Phenolic glycosides of forage legume *Onobrychis viciifolia*. Phytochemistry.

[22] Xu S., Duan W., Fang L., Sun Y., Wang X. (2012). Isolation and characterization of chemical constituents from the petals of *Nelumbo nucifera*. Asian J. Chem..

[23] Beninger C.W., Hosfield G.L. (1999). Flavonol glycosides from Montcalm dark red kidney bean: Implications for the genetics of seed coat color in *Phaseolus vulgaris* L.. J. Agric. Food Chem..

[24] Saito N., Toki K., Honda T., Tatsuzawa F. (2012). Floral pigments isolated from the sky-blue flowers of *Oxypetalum caeruleum*. Heterocycles.

[25] Hari Kishore P., Vijaya Bhaskar Reddy M., Gunasekar D., Marthanda Murthy M., Caux C., Bodo B. (2003). A new coumestan from *Tephrosia calophylla*. Chem. Pharm. Bull..

[26] Kirecci S.L., Simsek A., Yuksel A., Gurdal H., Gurbuz Z.G., Usanmaz S. (2014). Relevance of seminal plasma nitric oxide levels and the efficacy of SSRI treatment on lifelong premature ejaculation. Andrologia.

[27] Hirakura K., Morita M., Nakajima K., Sugama K., Takagi K., Niitsu K., Ikeya Y., Maruno M., Okada M. (1997). Phenolic glucosides from the root of *Pueraria lobata*. Phytochemistry.

[28] Fiorentino A., D’Abrosca B., Pacifico S., Mastellone C., Piscopo V., Monaco P. (2006). Spectroscopic identification and antioxidant activity of glucosylated carotenoid metabolites from *Cydonia vulgaris* fruits. J. Agric. Food. Chem..

[29] Zhang Q.A., Fan X.H., Zhang Z.Q., Wang Q., Wang J. (2012). Antioxidant for phenolic extracts from Semen Astragali Complanati. Nat. Prod. Res. Dev..

[30] Xiao A.Z., Wang Z., Gu S.C., Xin Y.M. (2004). The anti-aging effect of Semen Astragali Complanati. Flight Surg..

[31] Wei C.P., Zhou D.H. (2010). Study on anti-aging effects of flavonoids of Astragali Complanati on subacute aging mice induced by D-galactose. Chin. Gen. Pract..

[32] Panche A.N., Diwan A.D., Chandra S.R. (2016). Flavonoids: An overview. J. Nutr. Sci..

[33] Kim M.S., Pang G.C., Lee M.W. (1997). Flavonoids from the leaves of *Rubus coreanum*. Yakhak Hoeji.

[34] Cassidy A., Franz M., Rimm E.B. (2016). Dietary flavonoid intake and incidence of erectile dysfunction. Am. J. Clin. Nutr..

[35] Rangkadilok N., Sitthimonchai S., Worasuttayangkurn L., Mahidol C., Ruchirawat M., Satayavivad J. (2007). Evaluation of free radical scavenging and antityrosinase activities of standardized longan fruit extract. Food Chem. Toxicol..

[36] Chiou W.F., Lin J.J., Chen C.F. (1998). Andrographolide suppresses the expression of inducible nitric oxide synthase in macrophage and restores the vasoconstriction in rat aorta treated with lipopolysaccharide. Br. J. Pharmacol..

